# Proteome Remodeling in Response to Sulfur Limitation in “*Candidatus* Pelagibacter ubique”

**DOI:** 10.1128/mSystems.00068-16

**Published:** 2016-07-12

**Authors:** Daniel P. Smith, Carrie D. Nicora, Paul Carini, Mary S. Lipton, Angela D. Norbeck, Richard D. Smith, Stephen J. Giovannoni

**Affiliations:** a Department of Microbiology, Oregon State University, Corvallis, Oregon, USA; b Biological Sciences Division, Pacific Northwest National Laboratory, Richland, Washington, USA; University of Luxembourg

**Keywords:** SAR11, regulation, riboswitch, transcriptome

## Abstract

The alphaproteobacterium “*Candidatus* Pelagibacter ubique” strain HTCC1062 and most other members of the SAR11 clade lack genes for assimilatory sulfate reduction, making them dependent on organosulfur compounds that occur naturally in seawater. To investigate how these cells adapt to sulfur limitation, batch cultures were grown in defined medium containing either limiting or nonlimiting amounts of dimethylsulfoniopropionate (DMSP) as the sole sulfur source. Protein and mRNA expression were measured before, during, and after the transition from exponential growth to stationary phase. Two distinct responses were observed, one as DMSP became exhausted and another as the cells acclimated to a sulfur-limited environment. The first response was characterized by increased transcription and translation of all “*Ca*. Pelagibacter ubique” genes downstream from the previously confirmed *S*-adenosyl methionine (SAM) riboswitches *bhmT*, *mmuM*, and *metY*. The proteins encoded by these genes were up to 33 times more abundant as DMSP became limiting. Their predicted function is to shunt all available sulfur to methionine. The secondary response, observed during sulfur-limited stationary phase, was a 6- to 10-fold increase in the transcription of the heme *c* shuttle-encoding gene *ccmC* and two small genes of unknown function (*SAR11_1163* and *SAR11_1164*). This bacterium’s strategy for coping with sulfur stress appears to be intracellular redistribution to support methionine biosynthesis rather than increasing organosulfur import. Many of the genes and SAM riboswitches involved in this response are located in a hypervariable genome region (HVR). One of these HVR genes, *ordL*, is located downstream from a conserved motif that evidence suggests is a novel riboswitch.

**IMPORTANCE** “*Ca*. Pelagibacter ubique” is a key driver of marine biogeochemistry cycles and a model for understanding how minimal genomes evolved in free-living anucleate organisms. This study explores the unusual sulfur acquisition strategy that has evolved in these cells, which lack assimilatory sulfate reduction and instead rely on reduced sulfur compounds found in oxic marine environments to meet their cellular quotas. Our findings demonstrate that the sulfur acquisition systems are constitutively expressed but the enzymatic steps leading to the essential sulfur-containing amino acid methionine are regulated by a unique array of riboswitches and genes, many of which are encoded in a rapidly evolving genome region. These findings support mounting evidence that streamlined cells have evolved regulatory mechanisms that minimize transcriptional switching and, unexpectedly, localize essential sulfur acquisition genes in a genome region normally associated with adaption to environmental variation.

## Abstract (precis)

“*Ca*. Pelagibacter ubique” is a key driver of marine biogeochemistry cycles and a model for understanding how minimal genomes evolved in free-living anucleate organisms. This study explores the unusual sulfur acquisition strategy that has evolved in these cells, which lack assimilatory sulfate reduction and instead rely on reduced sulfur compounds found in oxic marine environments to meet their cellular quotas. Our findings demonstrate that the sulfur acquisition systems are constitutively expressed but the enzymatic steps leading to the essential sulfur-containing amino acid methionine are regulated by a unique array of riboswitches and genes, many of which are encoded in a rapidly evolving genome region. These findings support mounting evidence that streamlined cells have evolved regulatory mechanisms that minimize transcriptional switching and, unexpectedly, localize essential sulfur acquisition genes in a genome region normally associated with adaption to environmental variation.

## INTRODUCTION

“*Candidatus* Pelagibacter ubique” strain HTCC1062 is one of the few aerobic marine bacteria unable to incorporate sulfur from the readily available pool of dissolved sulfate (SO_4_^2−^), instead depending on reduced organic compounds, including methionine, dimethylsulfoniopropionate (DMSP), and methanethiol (MeSH), for sulfur (1, 2). Metabolic strategies such as this are hypothesized to have arisen in response to evolutionary pressure for reduction of genome size (3, 4). The tradeoff—an increased dependence on organosulfur compounds produced by other members of the plankton community—suggested that natural populations of “*Ca*. Pelagibacter ubique” might occasionally become sulfur limited.

Large quantities of DMSP are synthesized by marine algae, which use this compound for its antioxidant, osmotic, and predator deterrent properties (5 – 10). Lysis of algal cells maintains a 1 to 100 nM DMSP concentration in the euphotic zone (7, 11 – 13), with a turnover rate of 2 to 28 h (11, 14). Though not as abundant as SO_4_^2−^, the surface seawater concentrations of DMSP are theoretically more than sufficient to meet the sulfur requirements of marine microorganisms (1, 14 – 17). Assimilation of DMSP sulfur is common among heterotrophic bacterioplankton and preferred over SO_4_^2−^ (11, 18). Species smaller than 1 µm in diameter (including “*Ca*. Pelagibacter ubique”) account for 66 to 100% of DMSP consumption (14, 19, 20). Uptake studies in the natural environment revealed members of the “*Ca.* Pelagibacter” (21) and *Roseobacter* (11, 22) genera to be the primary consumers of DMSP. “*Ca.* Pelagibacter” isolates are known to degrade DMSP to methylthioacryloyl-coenzyme A (CoA), and degradation to MeSH has been theorized (23 – 25) and demonstrated (2), though the gene encoding the activity has not been identified.

In addition to the production of MeSH, “*Ca*. Pelagibacter ubique” also cleaves DMSP to dimethylsulfide (DMS), with the MeSH/DMS stoichiometry hypothesized to depend on intracellular DMSP concentrations (2). Genera such as *Roseobacter*, which are not dependent on DMSP sulfur, have similarly been found to utilize DMSP carbon while discarding the sulfur from this compound into the environment in the form of DMS (22, 26 – 30). Because atmospheric DMS originating from oceanic DMSP has been implicated in global climate change (31 – 34), determining the factors driving the metabolic fate of DMSP has become increasingly important.

Sulfur limitation has been studied in a variety of bacteria, including *Bacillus* (35, 36), *Brevibacterium* (37), *Pseudomonas* (38, 39), and *Synechocystis* (40) species. All of these species respond to sulfur limitation by upregulating sulfur import systems and cysteine synthesis pathways. The primary sulfur assimilation strategy among these species is acquiring sulfate and sulfonates from the environment for incorporation into cysteine. From cysteine, transsulfuration is employed to generate homocysteine, which in turn is methylated by MetH or MetE to produce methionine. Most of the genes upregulated by these species in response to sulfur limitation are absent from the genome of “*Ca*. Pelagibacter ubique,” including both *metH* and *metE*.

**FIG 1:**
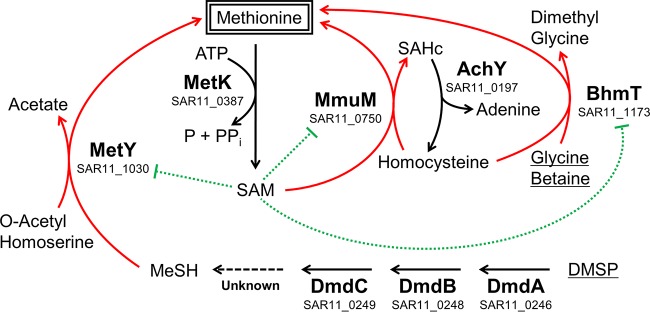
Products of genes with increased expression as DMSP becomes limiting. MetY, BhmT, and MmuM work independently to produce methionine, a key organic sulfur compound. Glycine betaine and DMSP are underlined to indicate their availability in the growth medium. Red lines indicate increased mRNA and/or protein expression in sulfur-limited exponential-phase samples (*n* = 3) relative to the expression in control exponential-phase samples (*n* = 4). Green lines denote the model for inhibition of translation by SAM-sensing riboswitches. DMSP, dimethylsulfoniopropionate; MeSH, methanethiol; SAHc, *S*-adenosyl homocysteine; SAM, *S*-adenosyl methionine.

Recent studies of DMSP catabolism have led to a greater understanding of how this abundant form of organic carbon and sulfur is utilized by marine bacterioplankton (24, 25, 28, 32, 41 – 43). The metabolic pathway used by “*Ca*. Pelagibacter ubique” to assimilate sulfur from DMSP into biomass begins with demethylation of DMSP by DmdA to yield methylmercaptopropionate (MMPA). The next three steps in this pathway are catalyzed by DmdB, DmdC, and DmdD to sequentially yield 3-methylmercaptopropionyl-CoA (MMPA-CoA), methylthioacryloyl-CoA (MTA-CoA), and acetaldehyde plus MeSH (Fig. 1). Because DmdD orthologs are absent from the “*Ca*. Pelagibacter ubique” genome, alternate enzymes are hypothesized to catalyze the final MeSH-generating step. The MetY or MetC enzyme completes the incorporation of DMSP-derived sulfur into biomass by condensing MeSH with *O*-acetyl homoserine to produce acetate and methionine (28, 44). Metabolic reconstruction from genome sequence data indicates that “*Ca*. Pelagibacter ubique” employs two additional pathways for methionine biosynthesis, in which methyl groups are transferred from glycine betaine to homocysteine by BhmT or from *S*-adenosyl methionine (SAM) to homocysteine by MmuM, in both cases forming methionine (45, 46).

In a wide variety of bacterial species, the metabolism of sulfur is regulated by riboswitches (47, 48). In this scheme, SAM binding domains present in the 5′ untranslated region (UTR) of mRNA inhibit the expression of downstream genes involved in sulfur metabolism when SAM concentrations are sufficient to meet cellular demands (49). In “*Ca*. Pelagibacter ubique,” SAM-V riboswitches that repress translation have been biochemically verified in the leader regions of *bhmT*, *mmuM*, and *metY* (50). Another class of SAM riboswitches that effect transcription of downstream genes, called SAM-II (51, 52), is present in the 5′ UTRs of *bhmT* and *metX*. A total of 16 characterized riboswitches and 27 loci with riboswitchlike characteristics have been computationally predicted in “*Ca*. Pelagibacter ubique” (53). The response of these genes to environmental stimuli, however, has yet to be determined with *in vitro* studies.

The atypical pathways for sulfur metabolism and proliferation of SAM-activated riboswitches in “*Ca*. Pelagibacter ubique” prompted us to study the changes in mRNA and protein expression in “*Ca*. Pelagibacter ubique” strain HTCC1062 in response to sulfur limitation. The observations we report support the conclusion that “*Ca*. Pelagibacter ubique” does not activate additional transporter genes for organosulfur acquisition when it becomes sulfur limited. Instead, transcription and translation increase in all genes located downstream from SAM-activated riboswitches, suggesting that the response to sulfur limitation is focused on increasing the concentrations of methionine-producing enzymes.

## RESULTS

Ten batch cultures of “*Ca*. Pelagibacter ubique” were grown in synthetic growth medium (54) and randomly selected to be amended with either 100 nM DMSP (sulfur limiting) or 1 µM DMSP (control) as the sole sulfur source (see Fig. S1 in the supplemental material). On average, the sulfur-limited cultures grew to a maximum cell density of 1.2 × 10^7^ cells/ml. Control cultures containing 10 times more DMSP grew to 3-times-higher densities, with an average maximum density of 3.5 × 10^7^ cells/ml. One control culture was excluded from analysis because its growth rate and maximum density were 1/2 and 1/10 those of the other control cultures. Each of the remaining nine cultures was harvested at three time points, (i) exponential growth phase, (ii) transitioning from exponential to stationary, and (iii) during late stationary phase. One control culture was harvested at two additional pre–stationary-phase time points to enable a more detailed temporal survey of gene expression. At each of the 29 time points, proteomic and microarray samples were collected simultaneously. After analyzing ribosomal protein mRNA expression patterns, the growth states of two samples classified as exponential phase were reclassified as transitioning and one transitioning sample as stationary phase.

10.1128/mSystems.00068-16.4 Figure S1  Growth curves and harvest time points for batch cultures. The densities of the nine cultures were measured once per day. Dots indicate time points at which samples were collected for proteomic and microarray analyses. Transcript abundances of ribosomal protein genes were used to classify samples as exponential phase, transitioning, or stationary phase, as detailed in Materials and Methods. Sample labels denote the group to which they were assigned, as follows: LE, sulfur-limited exponential phase; LS, sulfur-limited stationary phase; CE, control exponential phase; CS, control stationary phase. Download Figure S1, TIF file, 0.8 MB. Copyright © 2016 Smith et al. 2016 Smith et al. This content is distributed under the terms of the Creative Commons Attribution 4.0 International license.

### Initial response to sulfur depletion.

**FIG 2:**
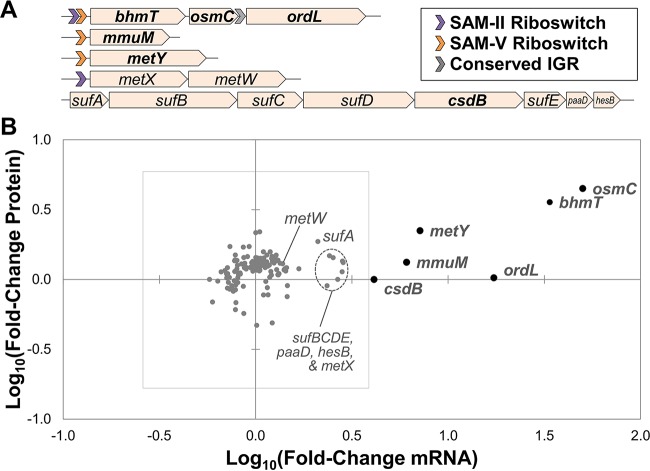
Exponential-phase differences between sulfur-limited and control cultures. Genes encoded downstream from *S*-adenosyl methionine (SAM) riboswitches were associated with higher mRNA and protein expression in sulfur-limited cultures. (A) Genomic loci associated with SAM riboswitches and/or higher expression. (B) All 134 genes whose expression was significantly different (*P* value of ≤0.05 for either mRNA or protein) between sulfur-limited exponential-phase samples (*n* = 3) and control exponential-phase samples (*n* = 4), plotted as log10(sulfur-limited abundance/control abundance) mRNA on the *x* axis and protein on the *y* axis. The inner box denotes a fivefold change; genes outside this threshold are in boldface in both panels.

Cultures were first harvested as their density reached 1 × 10^7^ cells/ml—near the maximum density of sulfur-limited cultures but well below the maximum density of control cultures. Comparing exponential-phase samples from sulfur-limited cultures (*n* = 3) to exponential-phase samples from control cultures (*n* = 4) revealed that remodeling of the transcriptome and proteome began prior to entering stationary phase. Cultures treated with a limiting concentration of DMSP showed higher levels of *osmC*, *bhmT*, *ordL*, *metY*, *mmuM*, and *csdB* mRNA transcripts, with correspondingly larger amounts of OsmC, BhmT, and MetY proteins (Table 1; Fig. 2).

**TABLE 1:** Comparison of differentially expressed mRNAs and proteins among all conditions^a^

Locus tag	%S^b^	Gene	Description	Fold change in expression under indicated condition of^c^:					
				mRNA			Protein		
				LE	LS	CS	LE	LS	CS
SAR11_0181	3.7	*ibpA*	Heat shock protein			**3.69**		3.74	**7.63**
SAR11_0254	1.3	*trmD*	tRNA methyltransferase		**0.51**	**0.44**			0.15
SAR11_0259	2.7		Hypothetical protein		**7.38**		ND	ND	ND
SAR11_0287	3.0	*ccmC*	Heme exporter membrane protein		**10.35**	**2.18**	ND	ND	ND
SAR11_0641	3.7	*recA*	Recombination protein		**7.31**	**3.11**		**2.65**	
SAR11_0750^d^	2.6	*mmuM*	Homocysteine *S*-methyltransferase	6.08	1.73	**5.22**			2.89
SAR11_1019	2.8	*xerD*	Integrase/recombinase		**8.50**	**7.63**	ND	ND	ND
SAR11_1030^d^	2.0	*metY*	*O*-Acetyl homoserine (thiol)-lyase	**7.14**	**6.37**	**9.78**	2.24	1.81	3.19
SAR11_1040	3.7	*hppA*	Proton-translocating pyrophosphatase		**0.12**	**0.27**			
SAR11_1093	3.8	*rpoA*	DNA-directed RNA polymerase		**0.13**	**0.42**	1.15	1.21	
SAR11_1102	2.2	*rplF*	Ribosomal protein L6		**0.16**	**0.26**			
SAR11_1104	5.0	*rpsN*	Ribosomal protein S14		**0.17**	**0.29**			
SAR11_1122	3.5	*rpoC*	DNA-directed RNA polymerase		**0.11**	**0.26**			
SAR11_1129	7.9		Hypothetical protein		**0.16**	**0.60**	ND	ND	ND
SAR11_1130	3.8	*tufB*	Translation elongation factor EF-Tu		**0.16**	**0.62**			
SAR11_1163	7.2		Hypothetical protein		**6.11**		ND	ND	ND
SAR11_1164	2.1		Hypothetical protein		**11.13**		ND	ND	ND
SAR11_1171^d^	2.0	*ordL*	Oxidoreductase	**17.28**	**4.88**	**11.29**			2.10
SAR11_1172^d^	1.6	*osmC*	Organic hydroperoxidase	**50.04**	**9.56**	**40.72**	4.48	10.42	2.16
SAR11_1173^d^	7.1	*bhmT*	Betaine-homocysteine *S*-methyltransferase	**33.77**	**8.91**	**29.19**	3.58		**6.20**
SAR11_1264	2.1	*metF*	Methylenetetrahydrofolate reductase		**0.20**	**0.12**	1.38	1.46	
SAR11_1265	4.4		Aminomethyltransferase		**0.10**	**0.10**	1.29	1.30	
SAR11_1274	2.9	*cspL*	Cold shock DNA-binding protein		**0.07**	**0.39**	1.58		0.78

a All 23 genes with significant differences (sixfold change and differential expression supported by a *P* value of less than or equal to 0.05) in mRNA or protein expression between control exponential-phase growth (*n* = 4) and any other condition are listed.

b %S, percentage of sulfur-containing amino acids.

c Values indicate the fold change in expression relative to the expression during control exponential-phase growth. Values are only displayed if the difference in expression is supported by a *P* value of ≤0.05 and are in boldface when the significance level is ≤0.05 after correcting for multiple comparisons. LE, sulfur-limited exponential phase; LS, sulfur-limited stationary phase; CS, control stationary phase; ND, not detected by mass spectrometry at any time point, potentially due to methodological limitations on extracting insoluble proteins, such as those localized to the membrane.

d Downstream from a SAM-V riboswitch.

### Stationary-phase differences.

Using the previously established sulfur requirement of 6.67 attomoles per “*Ca*. Pelagibacter ubique” cell (1), the concentration of DMSP unaccounted for by the biomass of late stationary-phase cultures was 39 nM (standard deviation [SD], ±5.5 nM) for the limited treatment and 789 nM (SD, ±31 nM) for the control treatment. The levels of messenger RNA transcripts for *ccmC*, *SAR11_1163*, and *SAR11_1164* were observed to be five- to eightfold higher in the sulfur-limited stationary-phase samples (*n* = 5) than in the control stationary-phase samples (*n* = 4) (Table 2). However, mass spectrometry was unable to identify peptides for these proteins at any time point. Descriptions of these three genes and an interpretation of their observed expression patterns are given in “Integral membrane proteins,” below.

**TABLE 2:** Effect of sulfur limitation on gene expression in stationary phase^a^

Locus tag	Gene	Description of product	Fold change (S-limited/control) in expression of^b^:	
			mRNA	Protein
SAR11_0007	*hflC*	Integral membrane proteinase	**0.20**	**1.34**
SAR11_0162	*groEL*	Chaperonin	**0.18**	
SAR11_0173		2-Hydroxy-6-oxo-2,4-heptadienoate hydrolase	**0.64**	1/∞
SAR11_0287	*ccmC*	Heme exporter membrane protein	**4.74**	ND
SAR11_0399	*Rbr*	Rubrerythrin; peroxidase	**0.17**	**2.24**
SAR11_0756	*aldA*	Acetaldehyde dehydrogenase	**0.23**	**0.63**
SAR11_0864		Hypothetical protein	**0.22**	**0.62**
SAR11_0865		Mannitol/chloroaromatic compound transport	**0.19**	
SAR11_1163		Hypothetical protein	**4.69**	ND
SAR11_1164		Hypothetical protein	**7.67**	ND
SAR11_1172^c^	*osmC*	Organic hydroperoxidase	**0.23**	**0.21**
SAR11_1274	*cspL*	Cold shock DNA-binding protein	**0.18**	1.27
SAR11_1302	*opuAC*	Glycine betaine ABC transporter: periplasmic	**0.22**	0.62
SAR11_1305	*glnT*	Glutamine synthetase	**0.19**	0.78
SAR11_1361	*livJ2*	Leu/Ile/Val-binding transport system	**0.23**	

a All 15 genes with fourfold or greater differences in mRNA or protein expression between sulfur-limited stationary-phase (*n* = 5) and control stationary-phase (*n* = 4) cultures are listed.

b Values greater than 1 indicate higher abundance in sulfur-limited condition. Differences in expression unsupported by a *P* value of 0.05 or less are omitted. Boldface indicates values that were significantly different (*P* ≤ 0.05) after correcting for multiple comparisons. ND, not detected by mass spectrometry at any time point, potentially due to methodological limitations on extracting insoluble proteins, such as those localized to the membrane; 1/∞, observed in sulfur-limited stationary samples but not detected in control stationary samples.

c Downstream from a SAM-V riboswitch.

### Correlation between mRNA and protein.

**FIG 3:**
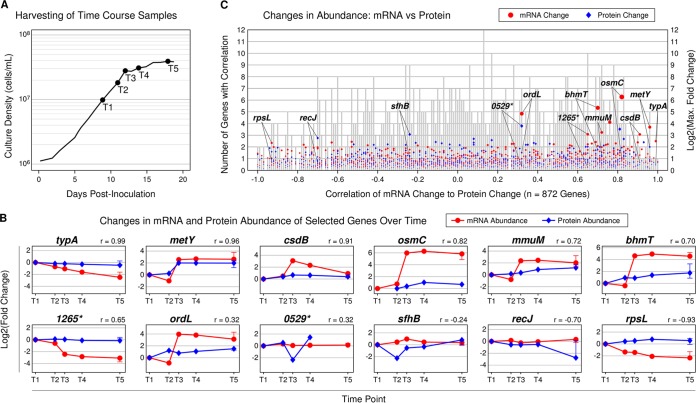
Magnitude of mRNA fold change is best predictor of mRNA-to-protein correlation. (A) Protein and mRNA abundances were analyzed at five time points (T1 to T5) from a single culture of “*Ca*. Pelagibacter ubique.” (B) Protein and mRNA expression of selected genes varied in correlation from −0.93 to +0.99. Error bars at T5 indicate the range of fold changes between the initial (exponential phase) and final (stationary phase) samples from all four control cultures. (C) Plotting all genes according to their protein-to-mRNA correlation throughout the five time points revealed that genes with particularly large changes in mRNA abundance (>eightfold, e.g., *osmC*) cluster near the high end of the correlation axis, indicating a trend between large mRNA changes and a corresponding change in protein. Point sizes are scaled by *y* axis position. The same 12 genes are highlighted in panels B and C. *, *0529* and *1265* are abbreviations for genes *SAR11_0529* and *SAR11*_*1265*.

Examining the relative expression levels of mRNA and protein in samples collected from the same culture across five time points revealed no systematic correlation between mRNA transcription and protein translation, with the exception that the most highly upregulated transcripts showed better correlation with the abundances of their protein products (Fig. 3C). The correlation coefficients for the genes were stochastically distributed in the range from −0.99 (inversely correlated) to 0.99 (directly correlated), indicating that the expression values were neither random (clustered near 0) nor interdependent (clustered near −1 or 1). This observation is in agreement with the pairwise comparisons of samples (Fig. 2B), in which the collective mRNA/protein ratios did not form a linear trend.

## DISCUSSION

The results of this experiment suggest a biphasic response to sulfur stress. Relative to control exponential-phase samples (*n* = 4), sulfur-limited exponential-phase samples (*n* = 3) had upregulated transcription and translation of methionine synthesis genes downstream from SAM-sensing riboswitches. Later, after 1 week in stationary phase, the transcript abundances of the heme *c* shuttle CcmC and two novel proteins were found to be higher in sulfur-limited stationary-phase samples (*n* = 5) than in control stationary-phase samples (*n* = 4). Interestingly, many of the genes induced in response to sulfur stress are encoded in a hypervariable region and are not found in closely related SAR11 strains. “*Ca*. Pelagibacter ubique” is distinct from other bacteria in that sulfur compound transporters were not upregulated in response to sulfur limitation—an observation that supports previous studies which postulated that organosulfur compounds are rarely, if ever, the limiting nutrient in marine environments (1, 14 – 17).

DMSP was selected for this study because it is widely distributed geographically, produced by a variety of plankton, and well described in the literature. Given the constitutive expression of transcripts and proteins related to organosulfur transport and the multiple upregulated pathways for methionine synthesis, the observations here may be representative of responses to limitation by other major sulfur sources. However, future studies are needed to test this hypothesis and explore the relative affinities of “*Ca*. Pelagibacter ubique” for different organosulfur nutrients.

### Riboswitches.

The four mRNA transcripts that were most upregulated in sulfur-limited exponential-phase samples (*n* = 3) relative to their levels in control exponential-phase samples (*n* = 4), *osmC*, *bhmT*, *metY*, and *mmuM*, are downstream from experimentally validated *S*-adenosyl methionine (SAM) riboswitches (50) (Fig. 2). These genes are all preceded by SAM-V class riboswitches that inhibit the translation of mRNA into protein by occluding the ribosome binding site when the concentration of SAM is plentiful; the *K_D_* (equilibrium dissociation constant) is 15 µM for *metY* and 120 µM for *bhmT* (50). One locus is also under the control of a SAM-II riboswitch, previously described as a regulator of methionine and cysteine metabolism in *Bacillus subtilis* and other Gram-positive bacteria (47, 48), which terminates transcription upstream from *bhmT* when intracellular SAM concentrations are sufficient; the *K_D_* for *bhmT* is 1.2 µM (50). Although tandem riboswitches are not uncommon, the SAM-II–SAM-V pairing is unique to “*Ca*. Pelagibacter ubique” among currently sequenced organisms and relatively rare in the global ocean survey metagenomic data set (53). A prior survey of the “*Ca*. Pelagibacter ubique” genome identified several intergenic regions similar to known riboswitches in length, GC content, and conservation (53), two of which are located immediately upstream from genes observed to increase in abundance in response to sulfur stress, *ordL* and *SAR11_1164*. Due to the close association between SAM riboswitches, mRNA expression, and protein expression, it is apparent that functional RNAs play a central role in this organism’s response to a low-sulfur environment.

The genes regulated by SAM-sensing riboswitches in “*Ca*. Pelagibacter ubique” are involved in the interconversion of the organosulfur compounds MeSH, methionine, SAM, homocysteine, and *S*-adenosyl-homocysteine (Fig. 1). Compared to their expression levels in the control exponential-phase samples (*n* = 4), three genes under the control of SAM-V riboswitches, encoding MetY, MmuM, and BhmT, were more highly expressed in the sulfur-limited exponential-phase samples (*n* = 3) as both mRNA and protein (Fig. 2). These three enzymes function independently to produce methionine (Fig. 1).

Counterintuitively, given the expectations for a sulfur limitation response, the abundance of MetK, ProX, and OpuAC, DmdA, DmdB, and DmdC proteins remained constant across all samples. MetK hydrolyzes ATP to convert methionine to SAM, in opposition to the highly upregulated MmuM, which catalyzes the reverse reaction. ProX, OpuAC, DmdA, DmdB, and DmdC import and degrade DMSP to MeSH, the organosulfur substrate for MetY. Accordingly, one might pose the question, “why would a sulfur-limited bacterium endlessly cycle methionine to and from SAM and not allocate more resources to importing sulfur-containing DMSP?” We speculate that “*Ca*. Pelagibacter ubique” has adapted to exploit DMSP resources in an environment where reduced sulfur is rarely limiting. Under this model, a decrease in SAM would indicate that the cell should draw on the abundant DMSP pool to make more methionine, thereby providing MetK with the substrate needed to generate more SAM. This riboswitch-mediated response is likely a homeostatic mechanism that maintains a constant intracellular supply of SAM by redistributing sulfur between different organosulfur molecules.

### Methionine and cysteine synthesis.

The second-most-upregulated gene after *osmC* is *bhmT*, encoding betaine-homocysteine methyltransferase. This enzyme catalyzes the demethylation of glycine betaine to dimethylglycine. The methyl group is transferred to homocysteine to form methionine. Two other upregulated genes also catalyze the synthesis of methionine: MmuM transfers a methyl group from SAM onto homocysteine to make methionine and *S*-adenosyl homocysteine, while one of the functions of MetY is to replace the acetyl group on *O*-acetyl homoserine with MeSH to form methionine and acetate (Fig. 1). The channeling of multiple organosulfur compounds into methionine appears to be the central strategy for “*Ca*. Pelagibacter ubique” when the pool of bioavailable reduced sulfur is nearing depletion.

Though common in eukaryotes, the *bhmT* gene is rarely found in bacteria. Another bacterium utilizing *bhmT* is the actinobacterium *Brevibacterium aurantiacum*, which in addition to MetY and other species-specific sulfur acquisition genes, upregulates BhmT (BL2496) during sulfur limitation (37). This conserved stimulus for *bhmT* between phyla, together with the gene’s 34-fold mRNA upregulation in “*Ca*. Pelagibacter ubique,” highlights the odd absence of *bhmT* from all but a few bacterial genomes. Interestingly, the *bhmT* gene from “*Ca*. Pelagibacter ubique” HTCC1062 is more closely related to orthologs in *Actinobacteria* and *Firmicutes* than to *bhmT* in “*Ca*. Pelagibacter ubique” strain HTCC7211 (see Fig. S2 in the supplemental material).

10.1128/mSystems.00068-16.5 Figure S2  The *bhmT* gene in “*Ca*. Pelagibacter ubique” shows evidence of horizontal gene transfer. The *bhmT* genes from the three “*Ca*. Pelagibacter ubique” strains HTCC1062, HTCC1002, and HTCC7211 do not form a single clade and are instead bisected by *bhmT* genes from *Actinobacteria* and *Firmicutes*. Beginning with the *bhmT* protein sequence from “*Ca*. Pelagibacter ubique” HTCC7211, 481 similar sequences were selected from NCBI’s nr database using 10 iterations of PSI-BLAST. These were aligned with MUSCLE, and a bootstrapped maximum-likelihood tree was constructed by invoking RAxML with the following parameters: -m PROTCATDAYHOFF -f a -# 500 -x 9078563412. Download Figure S2, TIF file, 0.4 MB. Copyright © 2016 Smith et al. 2016 Smith et al. This content is distributed under the terms of the Creative Commons Attribution 4.0 International license.

In contrast to genes for methionine biosynthesis, genes dedicated to cysteine biosynthesis, such as *cysK*, *cysE*, and *metC*, were not upregulated in response to sulfur stress. MetY and MetC in “*Ca*. Pelagibacter ubique” have high sequence similarity (E values of 6e−50 and 2e−45, respectively) to Rv1079, a gene in *Mycobacterium tuberculosis* that has been suggested to act as a cystathionine gamma-lyase (EC 4.4.1.1) to catalyze the reversible reaction from cystathionine to cysteine (55). Therefore, we hypothesize that MetY, in addition to synthesizing methionine, may also synthesize cysteine. However, the pathway by which cystathionine is generated in “*Ca*. Pelagibacter ubique” remains unclear. An alternative explanation for observing no difference in expression for cysteine synthesis proteins is that these genes may be constitutively expressed and are reliant on BhmT, MetY, and MmuM to maintain an adequate supply of methionine to be converted into cysteine.

### Assimilatory sulfate reduction genes.

Two of the proteins encoded in the “*Ca*. Pelagibacter ubique” genome that are implicated in sulfur metabolism are AprB and AprA (56). These subunits form the holoenzyme AprBA, which mediates the bidirectional transfer of sulfite onto AMP to form adenosine 5′-phosphosulfate (APS). In other microorganisms, this complex is a component of the assimilatory sulfate reduction pathway through which inorganic sulfur is incorporated into organic molecules. However, the absence of other enzymes needed for this pathway (*cysDNCHIJ*, *sat*, and *phsABC* genes) from the “*Ca*. Pelagibacter ubique” genome led prior investigations to conclude that “*Ca*. Pelagibacter ubique” is unable to utilize the assimilatory sulfate reduction pathway and might instead rely upon AprBA to detoxify sulfite accumulating in the cytoplasm as a by-product of organic sulfur compound degradation (1, 3). In support of this hypothesis, we observed *aprB* and *aprA* mRNA transcripts to be 3.13- and 5.88-fold lower in sulfur-limited stationary-phase samples (*n* = 5) than in control exponential-phase samples (*n* = 4). Furthermore, peptides for the AprA subunit were also slightly, though significantly (*P* = 0.00025) different between stationary-phase conditions, with a sulfur limited/control ratio of 6:5.

### OrdL regulation.

The oxidoreductase *ordL* stands out in this study for increasing 17-fold in transcript abundance while the abundance of its protein product did not change significantly. Although changes in transcriptomes are commonly not mirrored in proteomes, a discrepancy of this magnitude is unusual and therefore suggestive of a posttranscriptional control mechanism. A conserved 117-bp UTR has been previously noted in *ordL*’s upstream intergenic region (53), but no function or secondary structural fold was proposed for it. Our findings suggest that this 5′ UTR structure (see Fig. S3 in the supplemental material) might play a role in regulating *ordL* translation.

10.1128/mSystems.00068-16.6 Figure S3  Synteny, structure, and conservation of the *ordL* 5′ upstream intergenic region. (A) The genome context of *ordL* varies between the 10 available SAR11 strains. Gene numbering corresponds to the “*Ca*. Pelagibacter ubique” HTCC1062 homolog, or a gray placeholder is used where no HTCC1062 homolog has been identified. (B) The secondary structure of the intergenic region upstream from *ordL* in HTCC1062 was predicted using the Vienna RNAfold Web server (89) based on thermodynamic stability at 16°C. (C) HTCC1062 and four other “*Ca*. Pelagibacter ubique” genomes contained this intergenic region and exhibited a high degree of nucleotide conservation when aligned with MUSCLE (90). Download Figure S3, TIF file, 0.5 MB. Copyright © 2016 Smith et al. 2016 Smith et al. This content is distributed under the terms of the Creative Commons Attribution 4.0 International license.

The location of *ordL* immediately downstream from *bhmT* and *osmC* suggested that the biological roles of these three proteins might be related. OrdL belongs to a family of deaminating oxidoreductases that includes PuuB, an *Escherichia coli* enzyme that deaminates γ-l-glutamylputrescine to γ-glutamyl-γ-aminobutyraldehyde in the putrescine degradation pathway (57). However, the absence of other putrescine degradation pathway enzymes in the “*Ca*. Pelagibacter ubique” genome suggests that OrdL may be responsible for catalyzing a different reaction in this organism. The tertiary structure prediction program I-TASSER (58) identified structural similarities between “*Ca*. Pelagibacter ubique” OrdL and sarcosine oxidase motifs. This metabolic activity could allow OrdL to function analogously to BhmT, transferring methyl groups to homocysteine from an unknown donor molecule when glycine betaine is not present. We postulate that the conserved UTR upstream from OrdL is a riboswitch that binds to this unknown donor molecule, activating OrdL translation when this alternate methyl group donor is available. Alternatively, the BhmT reaction product dimethylglycine (DMG) may be a substrate for OrdL. If this interpretation of BhmT function is correct, we speculate that under sulfur-limiting conditions, DMG would be produced at a decreased rate and less OrdL would be required for metabolizing DMG; a DMG-sensing riboswitch could precisely regulate the translation of OrdL in response to fluctuations in DMG concentration and account for the discrepancy in OrdL expression observed in this study. While testing these models was beyond the scope of this study, they imply functions for a novel riboswitch that is likely to be a subject for further research.

### OsmC expression.

We observed upregulation of the “*Ca*. Pelagibacter ubique” OsmC protein. This protein is structurally similar to OsmC in *Escherichia coli*, which has been described as a peroxidase that favors organic hydroperoxides but also acts on inorganic hydrogen peroxide (59). As its name implies, *osmC* is induced by osmotic stress in *E. coli* and other species (60). The genome of “*Ca*. Pelagibacter ubique” also encodes the peroxidase rubrerythrin (*rbr*), but as Rbr relies on an iron-sulfur center (61 – 63), OsmC may be better suited to responding to the loss of osmolytes during low-sulfur conditions. A previous study of the proteome of “*Ca*. Pelagibacter ubique” in natural seawater medium also observed an increase in OsmC protein as cells entered stationary phase (64), an observation that was not repeated in limitation studies using iron (65) or nitrogen (66) as the limiting nutrient. Therefore, the expression of OsmC appears to be dependent on both the limiting condition and stationary-phase remodeling.

### Integral membrane proteins.

Previous studies have consistently found that bacterial responses to sulfur limitation involve increased production of sulfur compound transporters (36 – 38, 40), and iron limitation in “*Ca*. Pelagibacter ubique” was observed to result in a 27-fold increase in the periplasmic iron-binding protein SfuC (65). Therefore, it was unexpected that proteins for transporting organosulfur compounds were not observed at a higher abundance during sulfur-limited conditions, particularly the periplasmic binding components of the glycine betaine ABC transporters (ProX and OpuAC), which also bind and transport the organosulfur molecule DMSP (67).

The insoluble integral membrane protein CcmC has been well characterized as catalyzing the transfer of heme *c* groups to cytochrome *c* (68, 69). Because heme *c* molecules contain two sulfur atoms, they are likely to be less abundant in the cell during sulfur-limited conditions. It appears that the fivefold-higher expression of *ccmC* transcripts is a mechanism to compensate for this deficiency and maintain a constant supply of this essential cofactor to cytochrome *c*. The absence of peptide detections for CcmC is not unusual, as proteins such as CcmC are rarely detectable with the mass spectrometry sample preparation techniques used in this study.

Two proteins of unknown function, SAR11_1163 and SAR11_1164, were transcribed at levels 6 and 11 times higher, respectively, in sulfur-limited stationary-phase samples (*n* = 5) than in control stationary-phase samples (*n* = 4) (Table 2). The proteins encoded by these transcripts were never detected by mass spectrometry, indicating that these genes may be translationally inhibited or localized to the membrane. SAR11_1164 has previously been annotated as a putative lipoprotein due to a predicted transmembrane domain (3, 70). On the chromosome, *SAR11_1163* and *SAR11_1164* are set apart from neighboring genes by 248 nucleotides downstream and 445 nucleotides upstream. Intergenic distances of this size are particularly conspicuous given that the median intergenic spacer in “*Ca*. Pelagibacter ubique” is only 3 nucleotides, and the upstream region was noted by Meyer et al. as sharing characteristics of known riboswitches (53). Searching for homologs to these genes using amino acid alignment and structural alignment techniques revealed that both genes are unique to “*Ca*. Pelagibacter ubique” and, therefore, may represent a previously unknown class of proteins used to relieve sulfur stress. The search also revealed that they are regulated by a novel sulfur-related riboswitch.

### Genetic variability.

Hypervariable regions (HVRs) are sections of a genome having variable gene content between closely related strains, likely arising from horizontal gene transfer. These genomic loci commonly encode a high proportion of novel hypothetical proteins and proteins known to confer increased fitness in a particular environment (71). HVRs are prevalent in many microorganisms, often encoding nitrogen fixation, iron and sucrose uptake, toxin and antibiotic resistance, and other genes for adapting to specific environmental stressors (72). Studies of differences in the HVR gene contents of closely related strains of the dominant marine phototroph *Prochlorococcus* found that the presence of nitrogen and phosphate assimilation genes correlated well with the availability of those macronutrients in the environment (73 – 77). In members of the SAR11 clade, the presence of genes relating to the metabolism of phosphate (77, 78) and glucose (79) have similarly been correlated with environmental conditions.

Wilhelm et al. identified four “*Ca*. Pelagibacter ubique” HVRs by aligning the Global Ocean Sampling (80) metagenomic sequences to the “*Ca*. Pelagibacter ubique” HTCC1062 genome. Three of these HVRs (HVR1, HVR2, and HVR4) were characterized by a predominance of genes for cell surface properties, transport, and secretion (70). However, HVR3, comprised of *bhmT*, *osmC*, *ordL*, *SAR11_1163*, *SAR11_1164*, and seven other genes of unknown function, had a less apparent role. In this study, increased expression of the five above-named genes suggests that HVR3 is responsive to changes in the availability of sulfur.

Comparing the genome of the coastal Oregon isolate “*Ca*. Pelagibacter ubique” strain HTCC1062 used in this study to that of “*Ca*. Pelagibacter ubique” strain HTCC7211, isolated from the Sargasso Sea, revealed the extent of variability at this HVR. Absent from the Sargasso Sea strain were homologs for *osmC*, *SAR11_1163*, and *SAR11_1164*, encoding the peroxidase and novel proteins of unknown function. A 2009 metaproteomic study found SAR11 phosphate transporters to be the most abundant proteins in Sargasso Sea surface waters (81), consistent with the theory of phosphate-limited productivity in the North Atlantic Gyre. Differences in the commonly limiting nutrients of HTCC1062 and HTCC7211 may be reflected by the composition of their HVRs, furthering the hypothesis that *osmC*, *SAR11_1163*, and *SAR11_1164* provide an advantage to “*Ca*. Pelagibacter ubique” strains present in the phosphate- and nitrogen-rich coastal waters of the Pacific Northwest.

To assess whether similar variability exists among the genes for the acquisition of reduced sulfur compounds among SAR11 strains, we examined two complete SAR11 genomes (HTCC1062 and HTCC7211) and five incomplete SAR11 genomes (HTCC1002, HTCC9565, HIMB5, HIMB59, and HIMB114) for genes involved in sulfur metabolism, consistent with the observations reported above that the genes for sulfur acquisition are in HVR3. We observed considerable variability between strains (Table 3). Despite the presence of AprA in many strains, there was no evidence of complete operons for assimilatory sulfate reduction within any member of this group of organisms.

**TABLE 3:** Phylogenetic distribution of genes related to sulfur metabolism

Gene	No. of orthologues of indicated gene present in genome of^a^:						
	“*Candidatus* Pelagibacter ubique” strain:				*Alphaproteobacteria* sp. strain:		
	HTCC1002	HTCC1062	HTCC7211	HTCC9565	SAR11 HIMB114	SAR11 HIMB5	SAR11 HIMB59
*bhmT*	1	1	1			1	3
*metY*	1	1	1	1	1	1	2
*mmuM*	1	1	1		1	1	1
*ordL*	1	1	1				2
*osmC*	1	1					1
*ccmC*	1	1	1	1	1	1	1
*SAR11_1163*	1	1		1			
*SAR11_1164*	1	1		1			
*cysA*							
*cysC*	1	1	1	1		1	
*cysD*							1
*cysG*							
*cysH*							
*cysI*							
*cysJ*							
*cysN*			1				1
*cysQ*	1	1	1	1	1	1	1
*cysU*							
*cysW*							
*Sbp*							
*serA*	1	1	1	2	2	1	2
*serB*					1		
*serC*							
*metE*					1		
*metH*			1				
*aprA*	1	1	1	1		1	

a Empty cells indicate the absence of the gene.

### Concluding remarks.

Our findings demonstrate that sulfur acquisition systems in “*Ca*. Pelagibacter ubique” strain HTCC1062 are constitutively expressed but that enzymatic steps leading to the essential sulfur-containing amino acid methionine are regulated by a unique array of riboswitches and genes, many of which, surprisingly, are encoded in a rapidly evolving genome region. “*Ca*. Pelagibacter ubique” exhibits two distinct responses to sulfur limitation. The observations support the model that, during exponential phase in sulfur-limited cultures, SAM-sensing riboswitches increase the mRNA and protein expression of the genes *bhmT*, *metY*, and *mmuM*, which act to synthesize methionine via separate pathways (Fig. 1 and 3). As the sulfur supply becomes exhausted in sulfur-limited stationary phase, the aforementioned genes are repressed and transcripts for *ccmC*, *SAR11_1163*, and *SAR11_1164* are upregulated. Additional CcmC helps maintain a steady supply of sulfur-containing heme *c* to cytochrome *c*, while the two latter genes are hypothesized to be regulated by a novel sulfur-sensing riboswitch and localize to the cytoplasmic membrane.

This study was designed to provide a better understanding of SAR11 sulfur metabolism. “*Ca*. Pelagibacter ubique” is a key driver of marine biogeochemistry cycles and a model for understanding the cell biology of streamlined, free-living, anucleate organisms. A number of examples of successful planktonic microorganisms that have streamlined genomes have been described recently (82, 83), focusing attention on the importance of simpler cell models to microbial ecology (84).

The loss of genes for assimilatory sulfate reduction across the SAR11 clade, an apparent example of selection for genome simplicity, implies that these cells might periodically face the environmental stress of sulfur limitation. This raises the question, “have they evolved adaptations to compensate for stress?” To reconstruct unusual rearrangements of subcellular systems found in these successful minimalists, it has been necessary to apply functional genomics approaches, which provide insight when systems do not easily fit within known schemes of metabolic and regulatory organization (66). Unexpectedly, the response we observed was partially localized to a variable genome region that is rich in SAM riboswitches, including tandem riboswitch configurations, and also includes multiple motifs that likely represent new riboswitch types of unknown function. These findings are consistent with other reports of reduced transcriptional switching and further describe the preservation of posttranslational control mechanisms in streamlined genomes (82, 85). Organosulfur requirements and sulfur limitation responses in SAR11 represent an interesting case of the tradeoffs associated with genome reduction and now seem likely to be an interesting special case of how cells use hypervariable genome regions to adapt to variation across environmental regimes.

## MATERIALS AND METHODS

### Growth media and harvesting.

Artificial seawater (ASW) medium was made using previously established protocols (54). Water, salts, and metals were added to 10 20-liter polycarbonate carboys as detailed in Table S1 in the supplemental material and then autoclaved for 10 h. After cooling to room temperature, the carboys were sparged with CO_2_ for 20 h and brought up to 20 liters using sterile water to compensate for evaporation due to autoclaving. Vitamins and nutrients were added to each carboy from a stock solution containing either large or small amounts of DMSP and then sparged overnight on air while cooling to 16°C. The final nutrient concentrations were as follows: 10 µM glycine, 500 nM glycine betaine, 500 µM pyruvate, and 100 nM or 1 µM DMSP. The medium was then inoculated with “*Candidatus* Pelagibacter ubique” HTCC1062 (53) growing exponentially in low-sulfur medium. Following inoculation, the cultures were incubated at 16°C with constant air sparging.

10.1128/mSystems.00068-16.2 Table S1  Composition of artificial seawater medium. Product numbers are provided in place of chemical names where ultrapure reagents are necessary. Download Table S1, DOCX file, 0.1 MB. Copyright © 2016 Smith et al. 2016 Smith et al. This content is distributed under the terms of the Creative Commons Attribution 4.0 International license.

Culture growth was tracked daily by staining cells with SYBR green and counting on a Guava EasyCyte flow cytometer (86). Samples for microarray and proteomic analysis were taken from each culture at three time points: exponential growth, exponential-to-stationary transition, and stationary phase. At these time points, 5 × 10^10^ cells were removed to a separate vessel and amended with 10 mg chloramphenicol, 100 µl 500 mM EDTA, and 100 µl 100× Halt protease inhibitor cocktail (catalog number 78438; Thermo Scientific) per liter of culture. Tangential flow filtration against a Pellicon 2 mini-ultrafiltration module 30-K membrane (catalog number P2C030C01; Millipore) reduced the volume of culture to less than 150 ml, which was subsequently centrifuged for 1 h at 20,000 rpm and 0°C. The pelleted cells were resuspended in 1 ml Tris-EDTA and centrifuged again in a single 1.5-ml tube for 40 min at 40,000 rpm and 10°C. After decanting the supernatant, the pellet was stored at −80°C until proteomic analysis. In parallel, 80 ml of culture was collected by centrifugation (1 h at 20,000 rpm and 0°C) for microarray analyses. Pellets were resuspended in 1 ml of RNAprotect bacterial reagent (catalog number 76506; Qiagen) and then centrifuged again in a 1.5-ml tube for 40 min at 40,000 rpm and 0°C. After decanting the supernatant, microarray samples were placed at −80°C until analysis.

### Messenger RNA measurements.

Messenger RNA was processed using the same protocol described previously (66). Total RNA was extracted using an RNeasy MinElute cleanup kit (catalog number 74204; Qiagen) and then amplified using the MessageAmp II bacterial RNA amplification kit (catalog number AM1790; Ambion) with biotin-11-UTP (catalog number AM8451; Ambion) according to the MessageAmp “Improved Protocol” Handbook. Ten micrograms of amplified RNA per sample were hybridized to *Pelagibacter*-specific Affymetrix microarray chips.

### Proteome quantification.

Protein expression was measured using capillary liquid chromatography-mass spectrometry as previously described (66). Briefly, samples were sonicated and barocycled to lyse cells and then digested with trypsin. Peptide separation was performed using a high-performance liquid chromatography column in line with an LTQ (linear trap quadrupole) Orbitrap Velos mass spectrometer. High-resolution mass spectrometry spectra were collected from duplicate runs for each biological sample and matched to entries in a “*Ca*. Pelagibacter ubique” accurate mass and time tag database (87). The mass spectrometry proteomics data have been deposited to the ProteomeXchange Consortium via the PRIDE (88) partner repository with the data set accession numbers PXD003672 and 10.6019/PXD003672.

Fold changes in protein abundance were calculated as described previously (66) and are detailed in Text S1 in the supplemental material. Briefly, peptides from the same treatment and time point (defined in the next section) were averaged together and divided by the average from the exponential-phase control samples (*n* = 4) or other specified reference set, and then peptide fold changes were averaged together to arrive at a protein fold change.

10.1128/mSystems.00068-16.1 Text S1  Additional information detailing experimental protocols. Includes mRNA preparation, RNA labeling and microarray processing, mass spectrometry sample preparation, capillary liquid chromatography-mass spectrometry analysis, and calculation of protein abundance. Download Text S1, DOCX file, 0.1 MB. Copyright © 2016 Smith et al. 2016 Smith et al. This content is distributed under the terms of the Creative Commons Attribution 4.0 International license.

### Time point classification.

Harvest time points were selected to provide a minimum of 2 × 10^10^ cells for mass spectrometry. As a result, the sulfur-limited exponential-phase samples were harvested close to stationary phase. In order to ensure that samples categorized as exponential phase are biologically accurate, the levels of expression of *rpsCEGHJLNS*, *rplBCDEFNOPRVWX*, and *fusA* mRNA were taken into account. These 21 genes were selected because they are nearly contiguous loci of ribosomal proteins which all decreased significantly in mRNA expression (*P* < 0.01) between the exponential-phase control samples (*n* = 4; all ≤50% maximum cell density) and stationary-phase control samples (*n* = 4) marked in Fig. S1 in the supplemental material. The transcript RNA expression values from the microarray chips for each gene were first log_2_ transformed and then normalized to a range of 0.0 to 1.0. A sample’s “growth state” was then calculated by averaging all 21 genes’ normalized expression values in that sample. For example, a growth state of 1.0 would indicate that all 21 genes were expressed at their maximum observed abundances. The samples from this study clustered into three distinct growth states, 0.91 to 0.80, 0.58 to 0.39, and 0.29 to 0.08, which corresponded well to the exponential, transitioning, and stationary labels. This qualitative assessment reclassified two sulfur-limited exponential-phase samples as transitioning.

### Accession number.

 Microarray data collected by this study are available in the NCBI GEO database under accession number GSE31630.

10.1128/mSystems.00068-16.3 Data Set S1  Mass spectrometry, microarray, and flow cytometry data. Includes all experimentally observed abundance values used to create figures and tables in this article. Download Data Set S1, XLS file, 14.1 MB. Copyright © 2016 Smith et al. 2016 Smith et al. This content is distributed under the terms of the Creative Commons Attribution 4.0 International license.
