# Supplementary figures and images for: Proteome Remodeling in Response to Sulfur Limitation in “Candidatus Pelagibacter ubique”
Source: mSystems. 2016 Jul 12;1(4):e00068-16. doi: 10.1128/mSystems.00068-16 (PMC5069961; doi:10.1128/mSystems.00068-16)

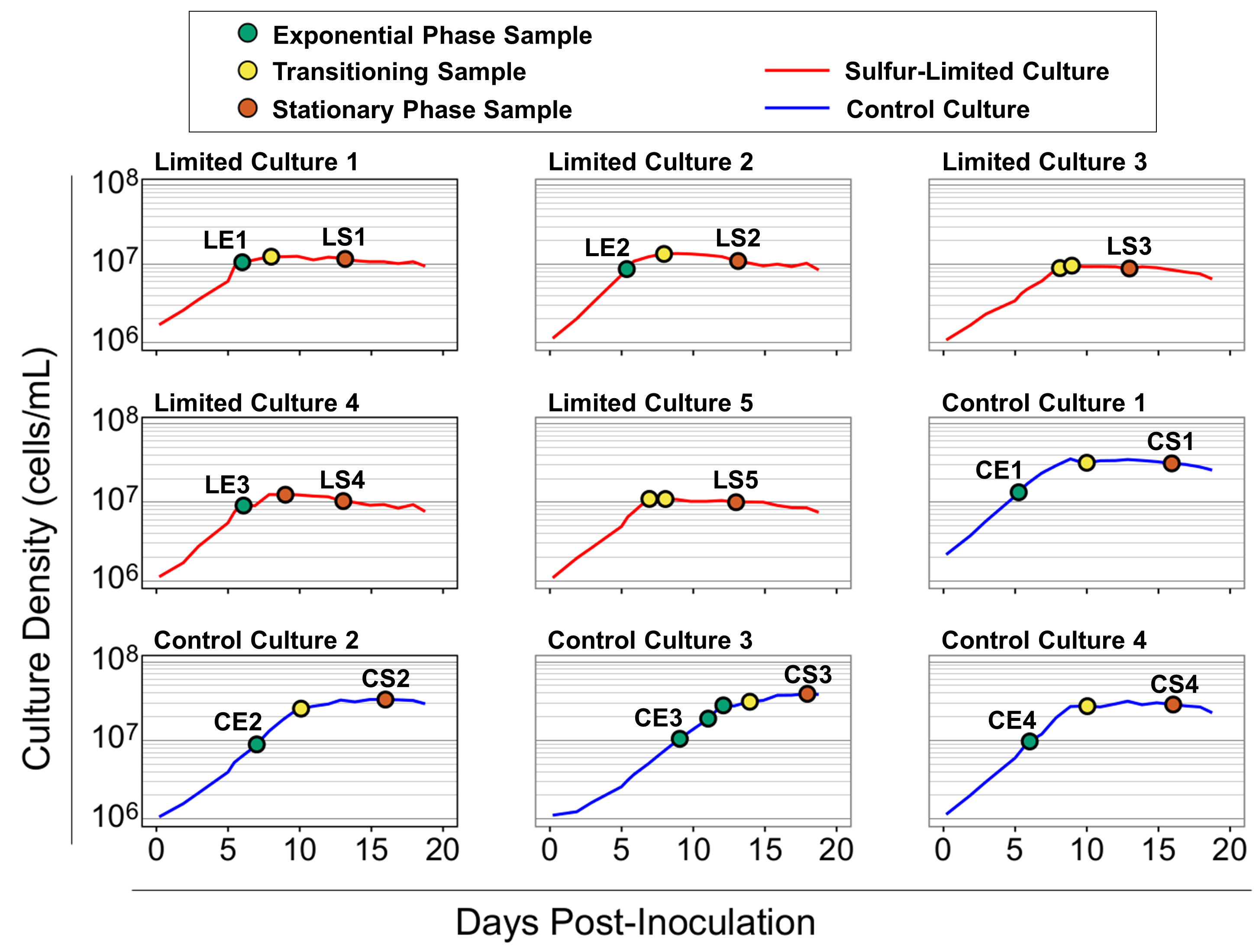

Supplement: Figure S1 [file sys004162036sf1.tif]

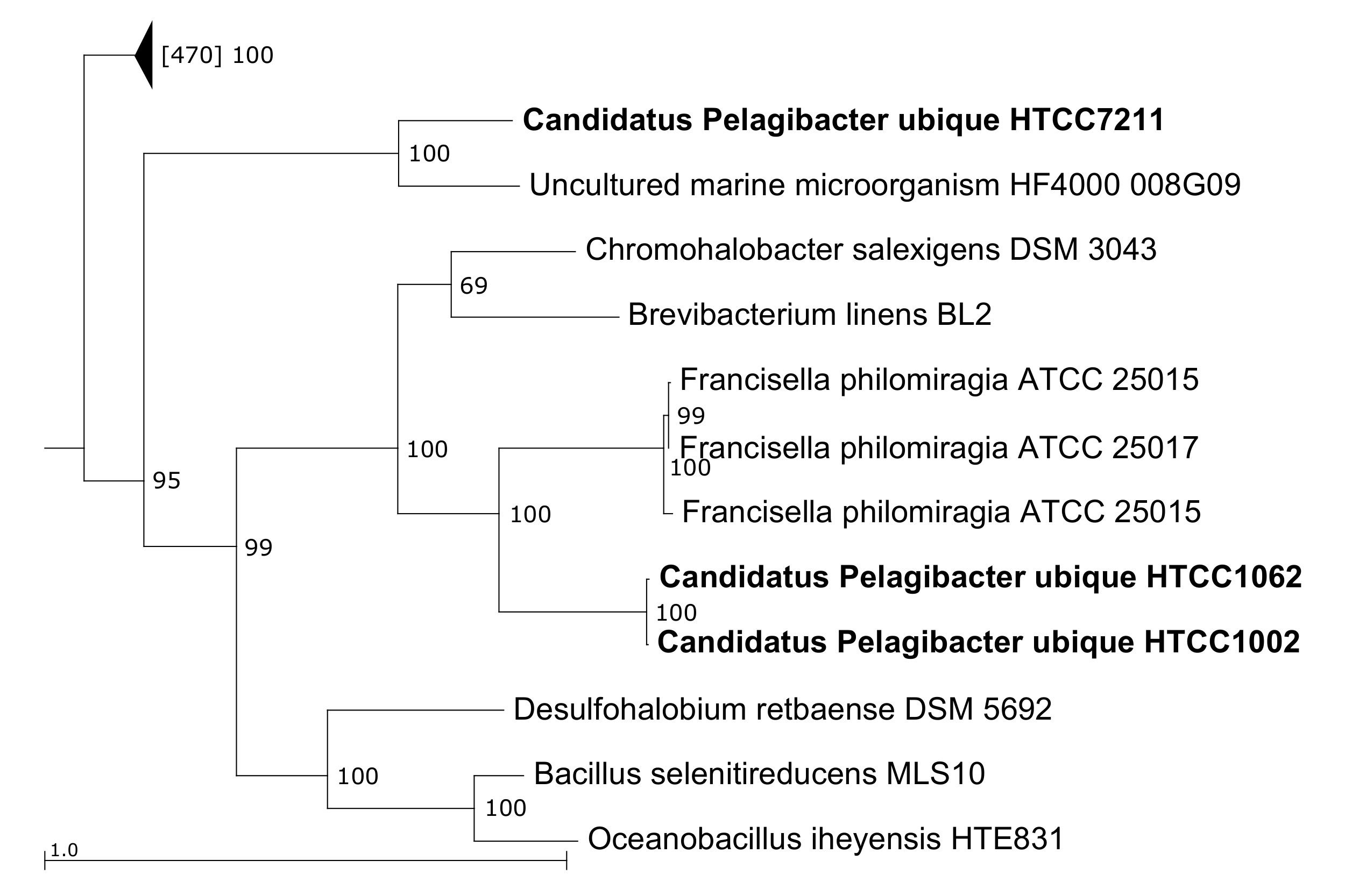

Supplement: Figure S2 [file sys004162036sf2.tif]

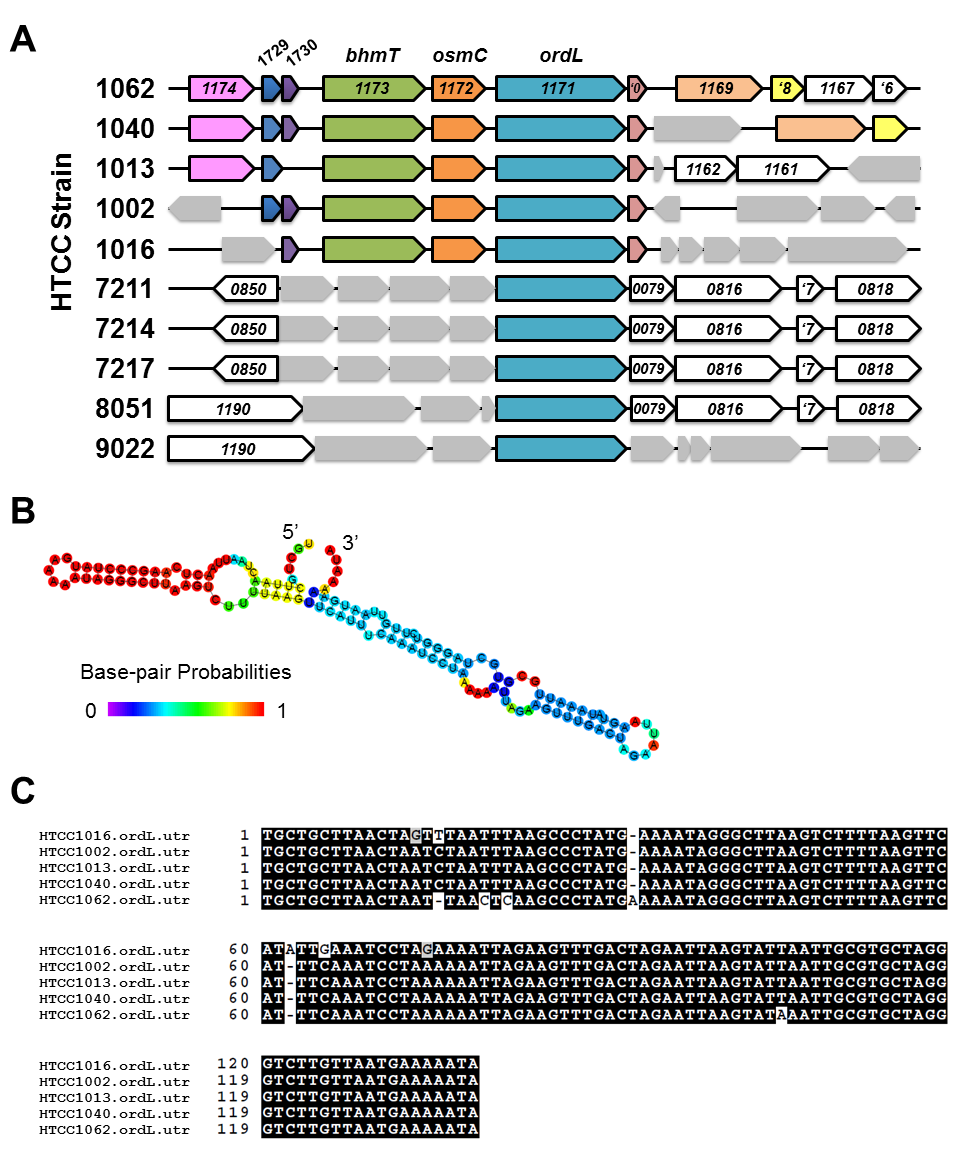

Supplement: Figure S3 [file sys004162036sf3.tif]
