# Supplementary material for: Proteome Remodeling in Response to Sulfur Limitation in “Candidatus Pelagibacter ubique”
Source: mSystems. 2016 Jul 12;1(4):e00068-16. doi: 10.1128/mSystems.00068-16 (PMC5069961; doi:10.1128/mSystems.00068-16)
Supplement: Table S1 [file sys004162036st4.docx]

**Table S1**

| Chemical Name | Formula | Molecular Weight | Sulfur Limited | Control |
| --- | --- | --- | --- | --- |
| DMSP | C_5_H_10_O_2_S•HBr | 214.1 | **100 nM** | **1 µM** |
| Glycine | C_2_H_5_NO_2_ | 75.1 | 10 µM | 10 µM |
| Glycine Betaine | C_5_H_11_NO_2_•H_2_O | 135.2 | 500 nM | 500 nM |
| Pyruvate | C_3_H_3_NaO_3_ | 110.0 | 500 µM | 500 µM |
| Ammonium | NH_4_Cl | 53.5 | 100 µM | 100 µM |
| Phosphate | NaH_2_PO_4_ | 120.0 | 50 µM | 50 µM |
| Sigma #S7653 | NaCl | 58.4 | 460 mM | 460 mM |
| Sigma #M5921 | MgSO_4_•7H_2_O | 246.5 | 28 mM | 28 mM |
| Sigma #M2670 | MgCl_2_•6H_2_O | 203.3 | 27 mM | 27 mM |
| Sigma #C5080 | CaCl_2_•2H_2_O | 147.0 | 10 mM | 10 mM |
| Sigma #P9333 | KCl | 74.6 | 9 mM | 9 mM |
| Sigma #S6297 | NaHCO_3_ | 84.0 | 2 mM | 2 mM |
| Sigma #P9881 | KBr | 119.0 | 800 µM | 800 µM |
| Baker 0084-01 | H_3_BO_3_ | 61.8 | 400 µM | 400 µM |
| Sigma #S0390 | SrCl_2_•6H_2_O | 266.6 | 91 µM | 91 µM |
| Sigma #S1504 | NaF | 42.0 | 68 µM | 68 µM |
| Iron | FeCl_3_•6H_2_O | 270.3 | 100 nM | 100 nM |
| Zinc | ZnSO_4_•7H_2_O | 287.5 | 100 nM | 100 nM |
| Molybdenum | Na_2_MoO_4_•2H_2_O | 241.9 | 100 nM | 100 nM |
| Copper | CuSO_4_•5H_2_O | 249.7 | 10 nM | 10 nM |
| Manganese | MnCl_2_•4H_2_O | 197.9 | 10 nM | 10 nM |
| Selenite | Na_2_SeO_3_ | 172.9 | 1 nM | 1 nM |
| Nickel | NiCl_2_•6H_2_O | 237.7 | 1 nM | 1 nM |
| Aluminum | AlK(SO_4_)_2_•12H2O | 474.4 | 1 nM | 1 nM |
| Cobalt | CoCl_2_•6H_2_O | 237.9 | 1 nM | 1 nM |
| Cadmium | CdCl_2_•2½H_2_O | 228.3 | 500 pM | 500 pM |
| myo-Inositol | C_6_H_12_O_6_ | 180.2 | 5 µM | 5 µM |
| Thiamine HCl | C_12_H_17_N_4_OS•ClHCl | 337.3 | 5 µM | 5 µM |
| Niacin | C_6_H_5_NO_2_ | 123.1 | 1 µM | 1 µM |
| Pantothenate | (C_9_H_16_NO_5_)_2_Ca | 476.5 | 1 µM | 1 µM |
| Pyridoxine HCl | C_8_H_11_NO_3_•HCl | 169.2 | 1 µM | 1 µM |
| PABA | C_7_H_7_NO_2_ | 137.1 | 100 nM | 100 nM |
| d-Biotin | C_10_H_16_N_2_O_3_S | 244.3 | 5 nM | 5 nM |
| Folic Acid | C_19_H_19_N_7_O_6_ | 441.4 | 5 nM | 5 nM |
| B12 | C_63_H_88_CoN_14_O_14_P | 1355.4 | 1 nM | 1 nM |
