# Supplementary material for: Proteome Remodeling in Response to Sulfur Limitation in “Candidatus Pelagibacter ubique”
Source: mSystems. 2016 Jul 12;1(4):e00068-16. doi: 10.1128/mSystems.00068-16 (PMC5069961; doi:10.1128/mSystems.00068-16)
Supplement: Text S1 [file sys004162036s5.docx]

# Supplemental Materials and Methods

## Messenger RNA Preparation

Each cell pellet was resuspended in 100 μL TE (1 mM Tris; 1 mM EDTA; pH 8) containing 40 μg lysozyme and incubated at room temperature for 5 minutes. Total RNA was extracted using an RNeasy MinElute Cleanup Kit (Qiagen #74204) according to the manufacturer's instructions, with the exception of an additional wash step with 700 μL Buffer RW1 (Qiagen #1053394) immediately prior to the prescribed washes with Buffer RPE. Eluted RNA was quantified using a NanoDrop spectrophotometer (Thermo Scientific ND-1000) and found to vary over the range of 103 – 794 ng. Thirty-seven nanograms of total RNA from each sample was amplified and labeled using a MessageAmp II-Bacteria RNA Amplification Kit (Ambion #AM1790) and biotin-11-UTP (Ambion #AM8451) according to the MessageAmp "Improved Protocol" Handbook. Polyadenylation, reverse transcription, and second strand synthesis reactions were carried out in half the recommended volumes; the in-vitro transcription reaction was performed in the recommended volume and incubated for 19 hours rather than 14. NanoDrop measurements of amplified RNA showed recovery of 21 – 91 μg per sample.

## RNA Labeling and Microarray Processing

Five micrograms of total RNA was used to generate biotinylated complementary RNA (cRNA) for each treatment group using the One-Cycle Target Labeling protocol (Affymetrix, Santa Clara, CA) from the GeneChip Expression Analysis Technical Manual (701021 Rev. 5). In short, isolated total RNA was checked for integrity and concentration using the RNA 6000 Nano LabChip kit on the Agilent Bioanalyzer 2100 (Agilent Technologies, Inc., Palo Alto, CA). Poly-A RNA control kit RNA and *Ca.* P. ubique RNA were reverse transcribed using a T7-(dT) 24 primer and Superscript II reverse transcriptase (Invitrogen, Carlsbad, CA) and double stranded cDNA was synthesized and purified with GeneChip® Sample Cleanup Modules (Affymetrix, Santa Clara, CA). Biotinylated cRNA was synthesized from the double stranded cDNA using T7 RNA polymerase and a biotin-conjugated pseudouridine containing nucleotide mixture provided in the IVT Labeling Kit (Affymetrix, Santa Clara, CA). Prior to hybridization, the cRNA was purified with GeneChip Sample Cleanup Modules (Affymetrix, Santa Clara, CA), and fragmented. Ten micrograms from each experimental sample along with Affymetrix prokaryotic hybridization controls were hybridized for 16 hours to *Ca.* P. ubique genome arrays (pubiquea) in an Affymetrix GeneChip® Hybridization Oven 640. Affymetrix GeneChip® Fluidics Station 450 was used to wash and stain the arrays with streptavidin-phycoerythrin (Molecular Probes, Eugene, OR), biotinylated anti-streptavidin (Vector Laboratories, Burlingame, CA) according to the standard antibody amplification protocol for prokaryotic targets. Arrays were scanned with an Affymetrix GeneChip Scanner 3000 at 570nm. The Affymetrix prokaryotic hybridization control kit and Poly-A RNA control kit were used to ensure efficiency of hybridization and cRNA amplification. All cRNA was synthesized at the same time. Hybridizations were conducted with one replicate of all times and treatments concurrently. Each array image was visually screened to discount for signal artifacts, scratches or debris. All data was deposited in the NCBI GEO database under accession number GSE31630.

## Mass Spectrometry Sample Preparation

Each pellet was brought up to 100 µl with 8 M urea (Sigma-Aldrich, St. Louis, MO) and sonicated in a water bath with ice until the pellet went into solution. The samples were briefly spun and transferred to PCT MicroTube barocycler pulse tubes with 100 µl caps (Pressure Biosciences Inc., South Easton, MA). The MicroTubes were placed in a MicroTube cartridge and barocycled for 10 cycles (20 seconds at 35,000 psi back down to ambient pressure for 10 seconds). All of the material was removed from the MicroTubes and transferred to 1.5 mL micro-centrifuge tubes. A Coomassie Plus (Thermo Scientific, Rockford, IL) assay was used to determine protein concentration. Dithiothreitol (DTT) was added to each sample at a concentration of 5 mM (Sigma-Aldrich, St. Louis, MO) and incubated at 60 ˚C for 1 hour. The samples were then diluted 10-fold with 100 mM NH_4_HCO_3_, and tryptic digestion (Promega, Madison, WI) was performed at a 1:50 (w/w) ratio with the addition of 1 mM CaCl_2_ to stabilize the trypsin and reduce autolysis. The sample was incubated for 3 hours and cleaned via C-18 solid phase extraction (Supelco, Bellefonte, PA). The samples were dried to 50 µl and assayed with Bicinchoninic acid (Thermo Scientific, Rockford, IL) to determine the final peptide concentration and vialed for MS analysis.

## Capillary LC-MS Analysis

The HPLC system consisted of a custom configuration of 65-mL Isco Model 65D syringe pumps (Isco, Inc., Lincoln, NE), 2-position Valco valves (Valco Instruments Co., Houston, TX), and a PAL autosampler (Leap Technologies, Carrboro, NC), allowing for fully automated sample analysis across four separate HPLC columns (1). Reversed-phase capillary HPLC columns were manufactured in-house by slurry packing 3-µm Jupiter C_18_ stationary phase (Phenomenex, Torrence, CA) into a 60-cm length of 360 µm o.d. x 75 µm i.d. fused silica capillary tubing (Polymicro Technologies Inc., Phoenix, AZ) using a 1-cm sol-gel frit (unpublished PNNL variation of (2)) for retention of the packing material. Mobile phase consisted of 0.1% formic acid in water (A) and 0.1% formic acid acetonitrile (B). The mobile phase was degassed by using an in-line Degassex Model DG4400 vacuum degasser (Phenomenex, Torrence, CA). The HPLC system was equilibrated at 10 kpsi with 100% mobile phase A, and then a mobile phase selection valve was switched 50 min after injection, which created a near-exponential gradient as mobile phase B displaced A in a 2.5 mL active mixer. A 40-cm length of 360 µm o.d. x 15 µm i.d. fused silica tubing was used to split ~17 µL/min of flow before it reached the injection valve (5 µL sample loop). The split flow controlled the gradient speed under conditions of constant pressure operation (10 kpsi). Flow through the capillary HPLC column when equilibrated to 100% mobile phase A was ~500 nL/min.

MS analysis was performed using a LTQ Orbitrap Velos ETD mass spectrometer (Thermo Scientific, San Jose, CA) outfitted with a custom electrospray ionization interface. Electrospray emitters were custom made using 150 um o.d. x 20 um i.d. chemically etched fused silica (3). The heated capillary temperature and spray voltage were 250 ºC and 2.2 kV, respectively. Data was acquired for 100 min, beginning 65 min after sample injection (15 min into gradient). Orbitrap spectra (AGC 1×10^6^) were collected from 400-2000 m/z at a resolution of 100k followed by data dependent ion trap CID MS/MS (collision energy 35%, AGC 1×10^4^) and orbitrap ETD MS/MS (activation time 100 ms, AGC 2×10^5^) of the six most abundant ions. A dynamic exclusion time of 60 sec was used to discriminate against previously analyzed ions.

## Calculation of Protein Abundance

Tandem MS spectra were collected from 99 datasets from a combination of Thermo Electron LTQ instruments and hybrid LTQ-Velos Orbitrap instruments (See Capillary LC-MS Analysis methods section). These MS/MS spectra were searched against a 2006 version of the *Candidatus* P. ubique strain HTCC1062 proteome (4). The search program SEQUEST (5) was used to identify the peptides, and the MSGF score (6) was used to select peptides with a 1% False Discovery Rate (FDR) for identification and population into the AMT database. All peptides were assigned a normalized reverse-phase LC elution time (7), and the combination of the elution time and accurate mass was matched to LC-MS features from the experimental datasets.

High-resolution MS spectra were collected from duplicate runs for each biological sample on a Thermo Electron LTQ-Velos Orbitrap instrument. The deisotoped monoisotopic mass (8) and elution times were matched to the peptides in the AMT tag database (9) to obtain an identification and the abundance from the MS feature was used in the quantification. The mass spectrometry proteomics data have been deposited to the ProteomeXchange Consortium via the PRIDE (10) partner repository with the dataset identifier PXD003672 and 10.6019/PXD003672.

Raw abundances for peptides were log_2_ transformed, normalized using EigenMS in which 5 biases were detected and removed, and outliers removed using the open source software tool DanteR (11). Finally, peptide abundances were mean centered then un-log_2_ transformed.

Each of the 29 biological samples were measured with quantitative mass spectrometry in duplicate, producing 58 unique analyses that were binned into six conditions according the method described in "Time Point Classification" in the main methods section. Calculating the difference in protein abundance between two conditions was a three step process. First, the average peptide abundance for each condition was calculated by averaging together the values for individual peptides across all samples assigned to a particular condition. Peptides with fewer than three observations in a condition were marked as "not observed" in that condition. Next, the peptide average from condition 1 was divided by the peptide average from condition 2, then log_10_ transformed. Finally, all log_10_ peptide ratios from the same protein were averaged together.

To represent the likelihood that a protein was equally abundant in both samples, the multiple peptide measurements were combined into a single statistic as previously described (12, 13). Briefly, p-values for individual peptides were calculated using a one-tailed Student’s t-test. A two-tailed Student’s t-test was not used because p-values reflecting a large increase would be indistinguishable from p-values reflecting a large decrease. Instead, peptides which changed in the opposite direction from the protein average were assigned a p-value of 1 for their one-tailed Student’s t-test. All peptide p-values for a single protein were then combined into a single chi-square statistic by using Fisher’s method with a Bonferroni correction:

$${}^{2}=-2\times\sum_{i=1}^{n} \ln\left( \left\{ \begin{aligned} n\times P_{i}, &n\times P_{i}<1 \\ 1, &n\times P_{i}\geq1 \end{aligned} \right. \right)$$

Where *n* is the total number of peptides for a given protein and *P_i_* is an individual peptide's p-value as determined by a Student's t-test. The χ^2^ value is then transformed into a p-value using a chi-square probability table and 2*n* degrees of freedom.

1. **Livesay EA**, **Tang K**, **Taylor BK**, **Buschbach MA**, **Hopkins DF**, **LaMarche BL**, **Zhao R**, **Shen Y**, **Orton DJ**, **Moore RJ**, **Kelly RT**, **Udseth HR**, **Smith RD**. 2008. Fully automated four-column capillary LC−MS system for maximizing throughput in proteomic analyses. Anal Chem **80**:294–302.

2. **Maiolica A**, **Borsotti D**, **Rappsilber J**. 2005. Self-made frits for nanoscale columns in proteomics. Proteomics **5**:3847–3850.

3. **Kelly RT**, **Page JS**, **Luo Q**, **Moore RJ**, **Orton DJ**, **Tang K**, **Smith RD**. 2006. Chemically etched open tubular and monolithic emitters for nanoelectrospray ionization mass spectrometry. Anal Chem **78**:7796–7801.

4. **Giovannoni SJ**, **Tripp HJ**, **Givan S**, **Podar M**, **Vergin KL**, **Baptista D**, **Bibbs L**, **Eads J**, **Richardson TH**, **Noordewier M**, **Rappé MS**, **Short JM**, **Carrington JC**, **Mathur EJ**. 2005. Genome streamlining in a cosmopolitan oceanic bacterium. Science **309**:1242–1245.

5. **Yates JR**, **Eng JK**, **McCormack AL**, **Schieltz D**. 1995. Method to correlate tandem mass spectra of modified peptides to amino acid sequences in the protein database. Anal Chem **67**:1426–1436.

6. **Kim S**, **Gupta N**, **Pevzner PA**. 2008. Spectral probabilities and generating functions of tandem mass spectra: a strike against decoy databases. J Proteome Res **7**:3354–3363.

7. **Petritis K**, **Kangas LJ**, **Ferguson PL**, **Anderson GA**, **Paša-Tolić L**, **Lipton MS**, **Auberry KJ**, **Strittmatter EF**, **Shen Y**, **Zhao R**, **Smith RD**. 2003. Use of artificial neural networks for the accurate prediction of peptide liquid chromatography elution times in proteome analyses. Anal Chem **75**:1039–1048.

8. **Jaitly N**, **Mayampurath A**, **Littlefield K**, **Adkins JN**, **Anderson GA**, **Smith RD**. 2009. Decon2LS: An open-source software package for automated processing and visualization of high resolution mass spectrometry data. BMC Bioinformatics **10**:87.

9. **Pasa-Tolić L**, **Masselon C**, **Barry RC**, **Shen Y**, **Smith RD**. 2004. Proteomic analyses using an accurate mass and time tag strategy. BioTechniques **37**:621–639.

10. **Vizcaíno JA**, **Csordas A**, **del-Toro N**, **Dianes JA**, **Griss J**, **Lavidas I**, **Mayer G**, **Perez-Riverol Y**, **Reisinger F**, **Ternent T**, **Xu Q-W**, **Wang R**, **Hermjakob H**. 2016. 2016 update of the PRIDE database and its related tools. Nucleic Acids Res **44**:D447–456.

11. **Taverner T**, **Karpievitch YV**, **Polpitiya AD**, **Brown JN**, **Dabney AR**, **Anderson GA**, **Smith RD**. 2012. DanteR: an extensible R-based tool for quantitative analysis of -omics data. Bioinformatics **28**:2404–2406.

12. **Smith DP**, **Kitner JB**, **Norbeck AD**, **Clauss TR**, **Lipton MS**, **Schwalbach MS**, **Steindler L**, **Nicora CD**, **Smith RD**, **Giovannoni SJ**. 2010. Transcriptional and translational regulatory responses to iron limitation in the globally distributed marine bacterium *Candidatus* Pelagibacter ubique. PLoS ONE **5**:e10487.

13. **Hess A**, **Iyer H**. 2007. Fisher’s combined p-value for detecting differentially expressed genes using Affymetrix expression arrays. BMC Genomics **8**:96.
